# Development of a droplet digital PCR assay to detect illicit glucocorticoid administration in bovine

**DOI:** 10.1371/journal.pone.0271613

**Published:** 2022-07-15

**Authors:** Sara Divari, Matteo Cuccato, Antonella Fanelli, Francesca Tiziana Cannizzo

**Affiliations:** Department of Veterinary Science, University of Turin, Grugliasco (TO), Italy; University of Bari, ITALY

## Abstract

Glucocorticoids are often used illegally in food-producing animals for the growth promotion of livestock animals. In accordance to official chemical methods for glucocorticoid detection, an animal is declared as non-compliant when a residue is identified in the sample. Neverthless, growth promoting molecules can often escape identification due to their rapid elimination or due to the use of non-detectable new generation drugs. Therefore, an indirect screening method able to detect the biological effect of long-term administration of low doses of dexamethasone and prednisolone on livestock has been developed to support official methods. As already described, *FKBP5* (FKBP prolyl isomerase 5) expression in bovine thymus is regulated by glucocorticoids, and this specific regulation can be exploited in an indirect screening assay. In the present study, male veal calves and young bulls were considered in three different trials in which estradiol, dexamethasone, and prednisolone were administered alone or in combination with Revalor-200 subcutaneous pellets. Thoracic thymus was sampled from all animals and molecular analysis was performed. A duplex droplet digital PCR assay with EvaGreen^®^ was employed to detect the target gene expression using absolute quantification. The developed droplet digital PCR assay was precise, showing intra- and inter-assay mean coefficient of variation values of about 6.16% and 3.17%, respectively. It was also highly specific (100%) with Youden’s index of 76.92% and 53.57% applied to veal calves and young bulls, respectively. The lowest detection limit in which the target gene expression level was kept constant, was 0.05 ng/μl of cDNA with 1 copies/μL and 0.5 copies/μL for target and reference gene, respectively. This study establishes the basis for using a digital PCR-based assay as an efficient test to identify animals illegally treated with glucocorticoids.

## Introduction

In international animal production, growth promoters are often used to improve the productive performance of the animals, increasing lean tissue deposition and nitrogen retention, and reducing fat accumulation. Their use is well established in countries such as the United States and Canada, while in the European Union (EU) is forbidden [1,2].

Natural or synthetic growth promoters, above all beta-agonists, glucocorticoids (GC), and sex hormones are widespread in this field. In the EU, despite GCs use is permitted for therapeutic purposes only [3], these compounds are often illegally administered due to their positive influence on food intake and animal welfare [4].

However, the pharmacological activities of GC could represent a risk for meat consumers if these substances are administered without control [5,6]. Therefore, an organized and efficient system of official monitoring is required. The number of illegally treated animals is probably underestimated in Europe and this is most likely due to the limits of the official laboratory techniques. The official methods currently performed for meat samples standard analyses are liquid chromatography with mass spectrometry (LC-MS/MS) or gas chromatography with mass spectrometry (GC-MS/MS). However, very low individual doses of different molecules and the combination with synthetic analogues of known growth promoters could prevent their detection [7]. Therefore, the development of new screening methods based in particular on indirect parameters is getting supported.

The Italian National Program for Residue Surveillance has included the histological test as a screening method, which allows qualitative measurements in target organs to detect alterations induced by growth promoters’ administration [8–10]. It was reported that prolonged administration of low doses of dexamethasone induces dose-dependent thymus atrophy in cattle of different breeds [11], but the absence of thymus atrophy in prednisolone-treated animals was also observed. In addition, low residue concentrations are difficult to detect in urine during the treatment, making the detection of illegal prednisolone administration very challenging [12].

For this reason, a transcriptomic approach was previously proposed [13] and clear transcriptomic changes were observed in peripheral blood mononuclear cells of young bulls treated with prednisolone. Subsequently, aberrant FKBP prolyl isomerase 5 (*FKBP5*) gene expression was reported in the thymus and skeletal muscle of veal calves and young bulls following treatment with prednisolone, allowing the development of an indirect screening test based on *FKBP5* expression regulation by GCs [14,15].

In previous studies [14,15], *FKBP5* expression was evaluated using quantitative real-time PCR (qPCR) which currently represents the method of choice for this analysis. Transcript measurement can be assessed through an absolute or relative quantification, but they require a standard curve or a calibrator, respectively [16]. Droplet digital PCR (ddPCR) is an innovative technology that may facilitate gene expression evaluation [17]. In fact, in contrast to qPCR, ddPCR is an end-point PCR and absolute quantification is performed without the need of a standard curve [18] or inter-run calibrator [19]. Moreover, this method is relatively insensitive to PCR inhibitors and more sensitive in the detection of rare targets [20]. This method uses a microfluidics technology for partitioning the sample into multiple micro-reactions of defined volume (droplets). In ddPCR experiments, the absolute concentration of the target of interest is determined by the number of copies per microliter of reaction, based on positive and negative droplets under the assumption of Poisson distribution [20].

In this study, a duplex ddPCR assay was developed and proposed as an indirect screening test to identify GC-treated bovines.

## Materials and methods

### Experimental design and sample collection

This study was conducted on thoracic thymus from bovines experimentally treated with GC and collected at a slaughterhouse. Three trials were conducted as previously described [14,21–23].

The experiment was authorized by the Italian Ministry of Health and the Ethics Committee of the University of Turin, according to Council Directive 86/609/EU [24] and successive modifications [25]; the carcasses of the treated cattle were appropriately destroyed.

Trial 1 comprised 21 Friesian male veal calves (about 5 months old). They were divided into three groups weekly treated as follows: group DEX1 (n = 6) with estradiol benzoate (Sigma-Aldrich, Milan, Italy), 5 mg/animal, *i*.*m*. for 6 wk in combination with dexamethasone (Desashock^®^, Fort Dodge Veterinaria, Olot, Spain), 0.40 mg day^-1^, per *os*, for the last 31 days of treatment; group PDN1 (n = 7) orally treated with prednisolone acetate (Novosterol^®^, Vetem S.p.A., Porto Empedocle, Italy), 15 mg day^-^1 for 31 days, and group K1 (n = 8) considered as control. Animals were slaughtered 3 days after the last treatment.

Eighteen Charolaise young bulls (17–22 months old) were used in trial 2 and they were randomly divided into three experimental groups: group DEX2 (n = 5) was orally treated with 0.70 mg day^-1^ of dexamethasone for 40 days; group PDN2 (n = 6) was orally treated with 15 mg day^-1^ of prednisolone acetate for 35 days; and group K2 (n = 6) was the control group. The cattle were slaughtered 6 days after the withdrawal of the drug.

In trial 3, 23 Friesian young bulls ranging from 13 to 20 months in age were divided into three groups as follows: group DEX3 (n  =  8) that was administered with 200 mg of trenbolone acetate and 20 mg of 17β-oestradiol (Revalor-200, Intervet, USA) as a subcutaneous implant for 89 days plus dexamethasone per *os* (0.7 mg day^-1^) for 40 days; group PDN3 (n  =  8) was treated with prednisolone acetate at 30 mg day^-1^ per os for 35 days; group K3 (*n*  =  7) was untreated. The animals were slaughtered 6 days after GC withdrawal.

Moreover, samples of thoracic thymus obtained from each animal at slaughterhouse were immediately frozen in liquid nitrogen and then stored at -80°C for molecular analyses as previously described [14,26].

A field study was conducted on 116 samples of thoracic thymus (72 from veal calves and 44 from young bulls) collected at slaughterhouses of Turin area from January to December 2021. The samples were immediately submerged in approximately 5 volumes of RNAlater stabilization solution (Thermo Fisher Scientific, Vilnius, Lithuania) and then stored at -80°C for molecular analyses.

### RNA extraction and reverse transcription

RNA from thymus samples was extracted using QIAzol lysis reagent (Qiagen, Hilden, Germany) according to the manufacturer’s protocol. The RNA purity and concentration were determined by ultraviolet−visible spectrophotometry and the RNA integrity was verified by an automated gel electrophoresis system (Experion Instrument, Bio-Rad, Hercules, CA). cDNA was synthesized from 1 μg of total RNA using the QuantiTect Reverse Transcription Kit (Qiagen, Hilden, Germany), which included a DNase reaction, according to the manufacturer’s protocol.

### Reference gene selection by qPCR

Hypoxanthine phosphoribosyl-transferase I (*HPRT1*), β-actin (*ACTB*), TATA box binding protein (*TBP*), succinate dehydrogenase complex, subunit A (*SDHA*) and hippocalcin-like protein 1 (*HPCAL1*) were chosen as candidate reference genes (RGs) for gene expression analysis in thymus of veal calves (trial 1) and young bulls (trial 2 and 3). Such genes were selected because they presented an expression comparable to *FKBP5* in bovine thymus. Primer sequences to amplify RGs and *FKBP5* were selected from the literature or designed using Primer-BLAST [27] (S1 Table).

RGs analysis was conducted in qPCR (CFX Connect Real-Time PCR Detection System, BioRad, Hercules, CA) choosing a gene whose expression is thought to be relatively stable. The RGs expression stability (M) was determined using the geNorm algorithm [28] and BioRad CFX Maestro 2.2 software. The lowest M value defines the most stable endogenous control gene whereas M value of 1.5 indicates the limit beyond which the gene analyzed cannot be used as RG.

qPCR experiments were performed on 8.5 ng of cDNAs using iTaq Universal SYBR Green Supermix (Bio-Rad) with the following thermal protocol: 95°C for 30 s and 40 cycles at 95°C for 5 s and at 60°C for 30 s. A melting curve (from 65°C to 95°C) was performed at the end of each run to detect the dissociation of the PCR products. The assay efficiency (E) for each set of oligonucleotides was performed using appropriate serial dilutions of a pooled sample and the slope of the standard curve was used to calculate the qPCR efficiency as follows [29]:

$$E=10^{\left(\frac{1}{\mathrm{slope}}\right)}-1.$$

An acceptable qPCR efficiency value must be between 90–110%.

### qPCR experiments

Relative quantification of target gene was performed through qPCR using Pfaffl method [29]. Briefly, the relative quantity of target gene mRNA was calculated using amplification efficiencies and the most stable RG (determined as previously described) was used for normalization. Target gene expression was normalized by the relative quantity of an inter-run calibrator and the target gene expression level was indicated as normalized expression as follows:

$$normalized\;expression=\frac{E_{FKBP5}^{{\Delta Cq}_{FKBP5}(inter-run\;calibrator-sample)}}{E_{RG}^{{\Delta Cq}_{RG}(inter-run\;calibrator-sample)}}$$

Normalized expression data calculated by qPCR were then correlated to the expression level results obtained by duplex ddPCR.

### ddPCR experiments

The best RG identified by qPCR analysis was also used to normalize the expression level of the target gene in ddPCR. In order to establish the best conditions to perform the *FKBP5* gene expression analysis in ddPCR, the optimal annealing temperature for both target gene and the selected RG primers was assessed throughout a gradient from 55–65°C using 100 nM primers concentration. After optimization, the PCR protocol was at 95°C for 5 min, 40 cycles at 95°C for 30 sec and at 59°C for 1 min with an overall ramp rate of 2°C/s and three final steps at 4°C for 5 min, 95°C for 5 min, and 4°C indefinite hold for dye stabilization.

ddPCR assay was performed both in simplex (one gene per well) and duplex form (both target gene and RG in the same well) using 8.5 ng of six cDNA samples analyzed in triplicate for both *FKBP5* and RG expression. QX200 ddPCR EvaGreen Supermix (BioRad) and BioRad’s QX200 ddPCR system were used according to the manufacturer’s protocol. In brief, 20 μl of PCR mix and 70 μl of droplet generation oil for EvaGreen (BioRad) were added into DG8^TM^Cartridge (Bio-Rad) and droplets were created using QX200 droplet generator (BioRad). Droplets were transferred to ddPCR plates (BioRad) and PCR was performed using C1000 Touch thermal cycler (BioRad). One hundred nanomolar of each primer set were used in simplex ddPCR experiments. For duplex ddPCR assays, 50 and 150 nM of *FKBP5* and RG primers were used respectively to increase the differences in amplitudes between the two amplicons.

Intra- and inter-assay precision were calculated on duplex ddPCR experiments using six cDNA samples in triplicate. The analysis was repeated three times in three days and by three different operators and coefficients of variation (CV) were calculated. The expression level of *FKBP5* in each sample was determined as the ratio of target over RG copies/μl and every experimental group was compared to relative control.

The linearity of the duplex ddPCR assay was assessed on ten serial dilutions of target cDNA in triplicate and the detection limit for *FKBP5* and RG was calculated. In particular, the assay limit of detection was identified as the lowest concentration of cDNA and corresponding *FKBP5* and RG copies/μl, in which the expression level of the target gene was kept constant. Therefore the expression levels across the ten serial dilutions were compared.

To evaluate the accuracy of this ddPCR assay the Receiver Operating Characteristic (ROC) curve analysis was performed. This method allows the assessment of test sensitivity and specificity, which are true positive rate (GC-treated animals) and true negative rate (untreated animals), respectively. These parameters, together with Youden’s J index and the area under the curve (AUC), provide an adequate evaluation of the test performance.

Youden’s J index merges sensitivity and specificity (sensitivity + specificity—1) and the higher value shows the maximum potential of the assay which corresponds to a particular decision threshold or criterion values.

The ROC curves were constructed on *FKBP5*/RG values obtained from the experimental groups of trial 1 (n = 21, veal calves GC treated *vs* untreated) and trials 2 and 3 (n = 40, young bulls GC treated *vs* untreated). Youden’s index was also calculated with a 95% confidence interval. To verify the test applicability, a previously mentioned field study was conducted and mean ± standard deviation values of the copies/μl of *FKBP5* and RG transcripts were determined.

### Statistical analysis

All analyses were carried out with GraphPad prism 6 (version 6.07) (GraphPad Software, California, USA). Correlation analysis between qPCR and ddPCR experiments and between simplex and duplex ddPCR were conducted using the Pearson’s method. The linearity of the ddPCR assay was determined using linear regression analysis between triplicate of dilution series and their concentration (copies/μl). Friedman test was used to verify that expression levels across the cDNA dilution were statistically comparable. Due to the small number of samples, Kruskal-Wallis test was performed to detect significant gene expression differences between the experimental and control group of each trial. Statistical significance was set at *P*<0.05.

## Results

### Reference gene (RG) selection

The PCR amplification efficiency (S2 Table) and the gene expression stability (S1 Fig) of 5 candidate RGs were assessed in thoracic thymus samples from both veal calves (trial 1) and young bulls (trial 2 and 3) by qPCR. *HPRT1*, *ACTB* and *TBP* showed an optimal efficiency of reaction between 90–110% in all trials as the target gene *FKBP5*. Moreover, *HPRT1* and *TBP* were the most stable RGs in all trials. Indeed, their expression was less influenced by different experimental conditions. The stability parameter M values of *HPRT1* and *TBP* were 0.182 and 0.318 in trial 1 and trials 2–3 respectively. Taken together, these results indicated that *TBP* represented the best RG for normalization. In addition, the amplicon length was suitable for a duplex assay in ddPCR and its expression level (Cq) was comparable to *FKBP5* gene (S2 Table).

### qPCR experiments

**Fig 1:**
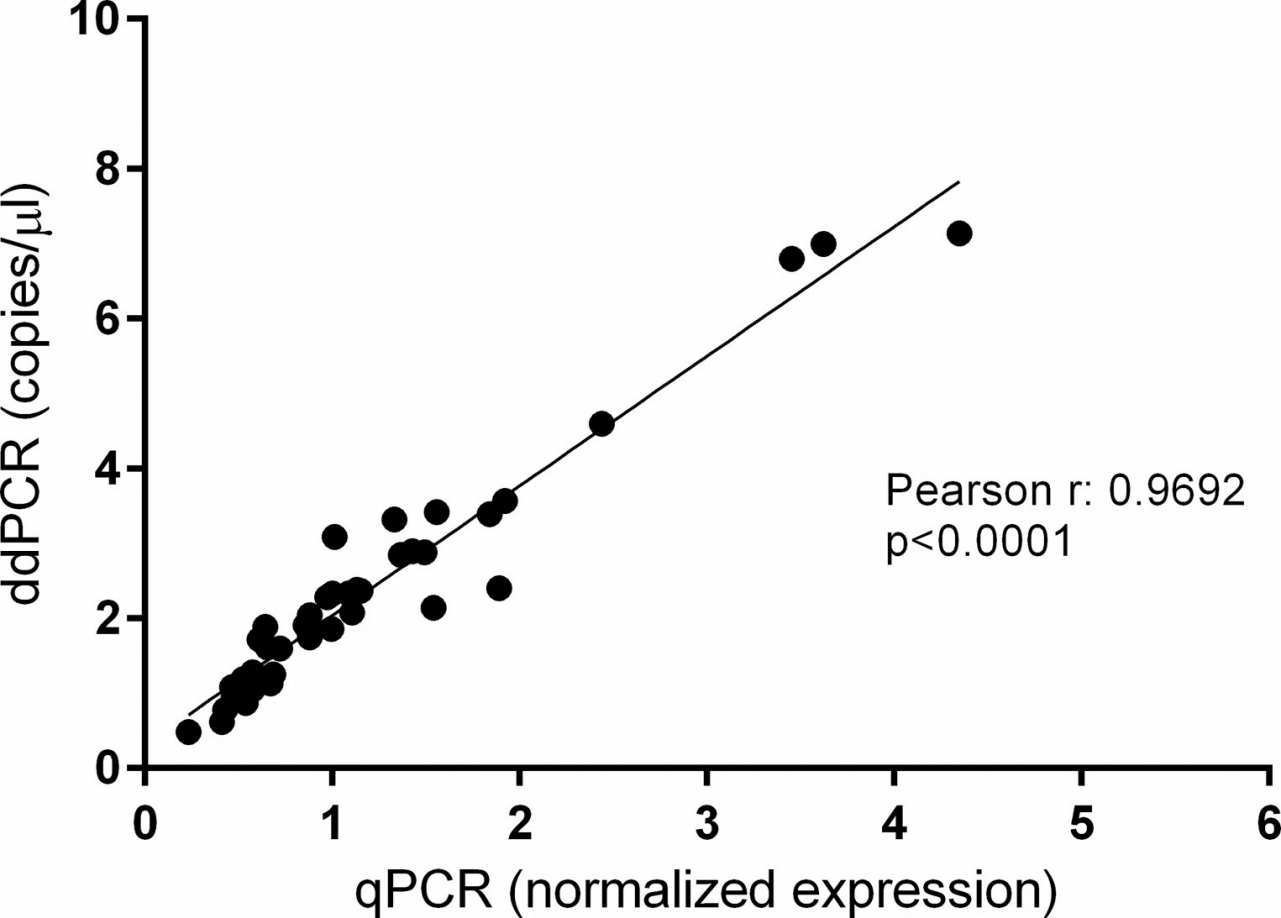
Correlation between qPCR and duplex ddPCR measurements. qPCR and ddPCR data were represented as normalized expression level of *FKBP5* and ratio of *FKBP5* and *TBP* (copies/μl) respectively. Each dot represents a sample of trials.

*FKBP5* gene expression levels obtained through qPCR were correlated to the expression levels obtained using duplex ddPCR. The two tests were significantly correlated (p<0.0001) with Pearson’s r of 0.9692 (Fig 1).

### Multiplexed *FKBP5* and *TBP* detection by ddPCR

**Fig 2:**
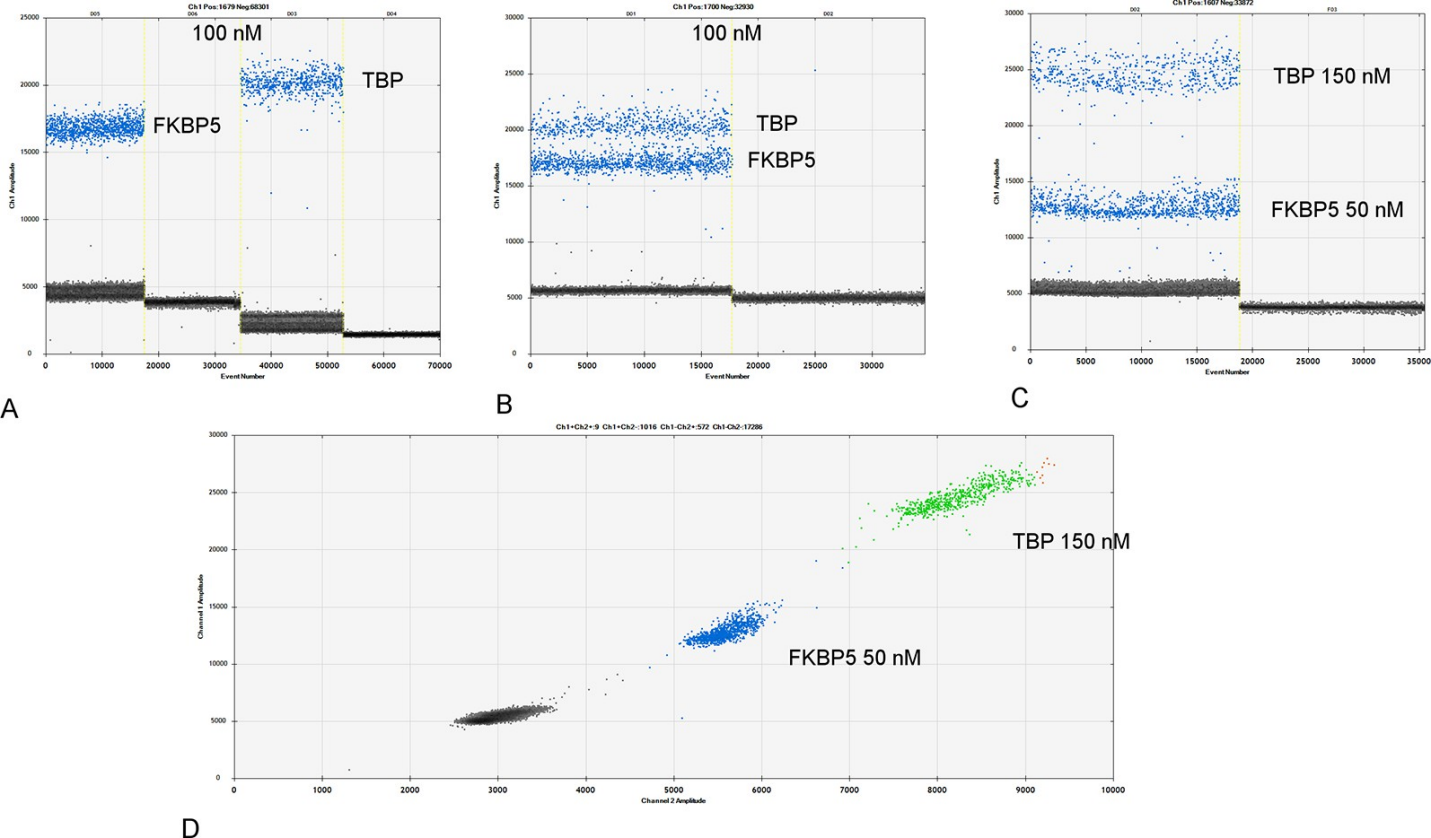
Simplex and duplex ddPCR assays. 1D droplet plot of simplex ddPCR assay to measure *FKBP5* and *TBP* gene expression (A). 1D droplet plot of duplex ddPCR assay combining the *FKBP5* and *TBP* assays into a single reaction and employing primer concentrations of 100 nM (B), and 50 nM and 150 nM respectively (C). 2D droplet plot of the *FKBP5/TBP* duplex assay (D) based on different primer concentrations. Blue, green and black dots represent *FKBP5*, *TBP* amplicons and negative droplets respectively. Orange dots correspond to droplets containing both gene amplicons.

PCR conditions were optimized as described in “materials and methods” section (S2 Fig). An annealing temperature of 59°C was chosen throughout a thermal gradient of 55–65°C for both *FKBP5* and *TBP* genes (Fig 2A). Distinction in droplet amplitude due to differences in *FKBP5* and *TBP* amplicon length was used to develop a duplex assay using EvaGreen^®^ technology (Fig 2B). The differences in amplitudes between the two amplicons were increased by adopting different primer concentrations for the two primer sets (Fig 2C and 2D).

**Fig 3:**
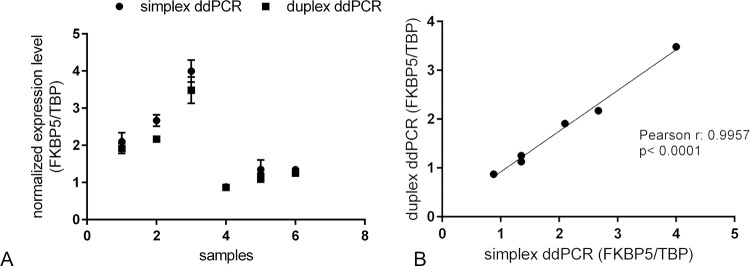
Normalized expression level of target gene. (A) *FKBP5* gene expression was detected in six samples of the bovine thymus by means of simplex and duplex ddPCR assay using EvaGreen dye. Error bars indicate the standard deviation of the measure triplicate. (B) Correlation analysis between simplex and duplex ddPCR measurements. The results are presented as the ratio of copies/μl of *FKBP5* and *TBP*.

The correlation between simplex and duplex assays was significantly high (p<0.0001) with Pearson r = 0.9957: the results were expressed as normalized expression (ratio between *FKBP5* copies/μl and *TBP* copies/μl) (Fig 3). Intra- and inter-assay values for duplex ddPCR are reported in Table 1 and the mean intra-run and inter-run CV% were 6.16% and 3.17% respectively.

**Table 1:** Intra-assay (test precision) and inter-assay variation (test variability) of duplex ddPCR using six samples of cDNA as start template.

Samples ID	Intra-assay		Inter-assay	
	FKBP5/TBP mean	CV%	FKBP5/TBP mean	CV%
1	1.91	6.97	1.93	2.14
2	2.17	3.18	2.14	1.24
3	3.48	10.18	3.42	2.38
4	0.87	3.92	0.86	3.44
5	1.13	11.04	1.19	6.02
6	1.25	1.70	1.26	3.83

Linearity of the assay was maintained between 0.05–25 ng/μl of cDNA and within the working interval of about 1–292 copies/μl and 0.5–170 copies/μl in reaction for *FKBP5* and *TBP* respectively (r^2^ FKBP5: 0.99, p<0.001; r^2^ TBP: 0.9983, p<0.001) (S3 Table). Specifically, between 0.05–25 ng/μl of cDNA, the expression level of the *FKBP5* normalized to *TBP* was not affected by cDNA concentration and the ratio values across the ten dilutions were not significantly different (p-value: 0.0743). Therefore, the lowest detection limit in which the expression level of the target gene was kept constant, was 0.05 ng/μl of cDNA with 1 copies/μL and 0.5 copies/μL for *FKBP5* and *TBP* respectively (S3 Fig).

The in-field study confirmed the calculated working range: in fact, 98.3% (114/116) and 100% (116/116) of samples collected and analyzed showed *FKBP5* and *TBP* gene copies/μl values within the range (S4 Fig).

### Gene expression regulation and ROC parameters of ddPCR assay

Normalized *FKBP5* gene expression was calculated in each trial using duplex ddPCR assay and it is reported in supplementary materials (S5 Fig). In particular, the ddPCR assay applied to trial 1 samples (S5A Fig) showed a significant down-regulation (*P*<0.001) of *FKBP5* (-2.56 fold) in groups treated with dexamethasone compared to control. In trial 2 (S5B Fig), dexamethasone and prednisolone induced a significant down-expression of *FKBP5* gene of about 4.32 (p<0.01) and 3.49-fold (p<0.05) compared to the control group. No statistically significant downregulation of *FKBP5* was detected using ddPCR in DEX3 and PDN3 groups of trial 3 (S5C Fig).

**Fig 4:**
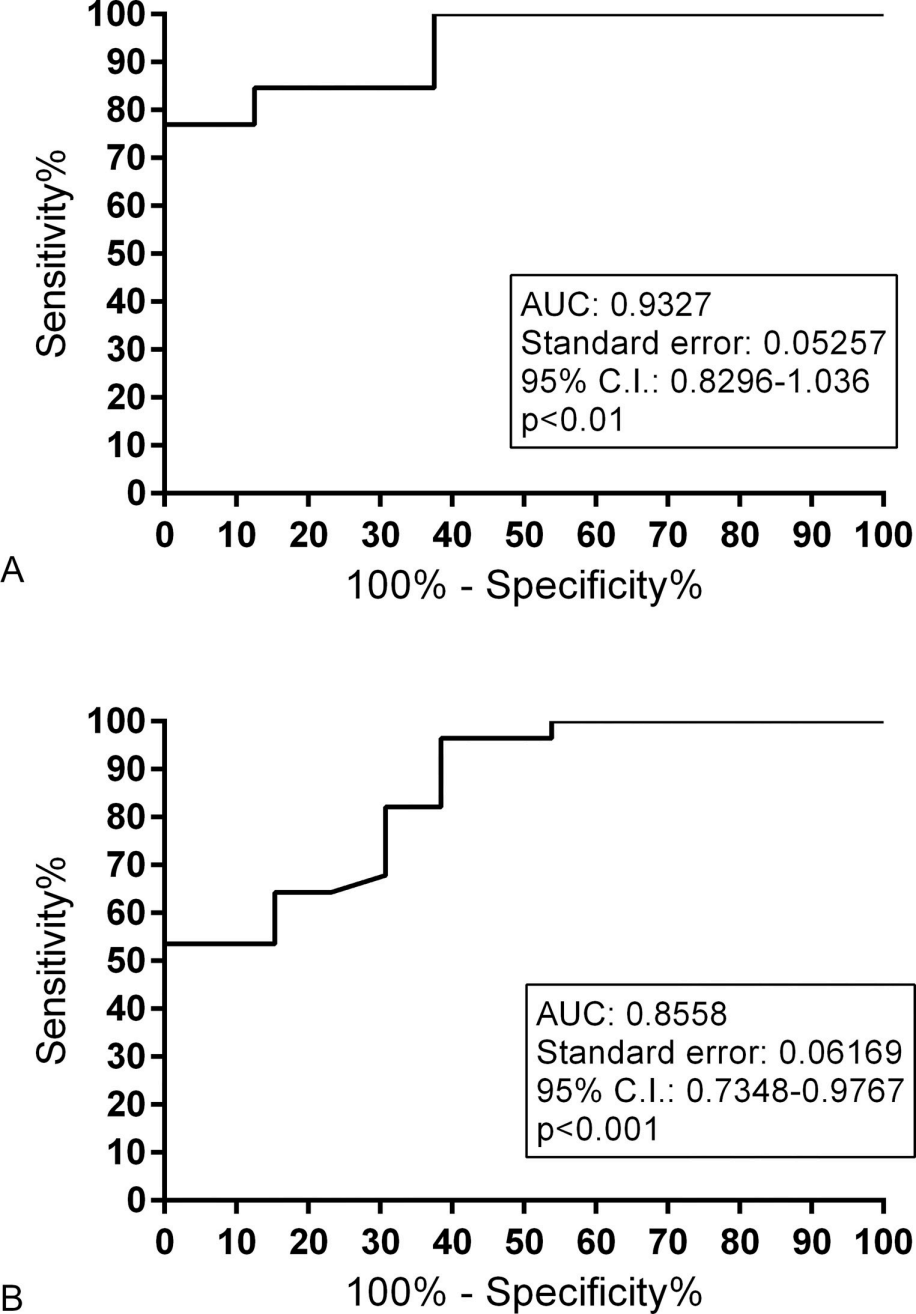
ROC curve. ROC curve for *FKBP5* down-regulation GC treated veal calves (A) and young bulls (B) versus untreated control animals (trial 1 and trials 2 and 3 respectively).

ROC curve related to veal calves GC treated compared to control (trial 1) showed the AUC of 0.9327, with a 95% confidence interval (0.8296–1.036; p < 0.01) (Fig 4A) and the ROC curve that describes the GC-treated young bulls compared to untreated animals of trial 2 and 3, presented AUC of 0.8558 with a 95% confidence interval (0.7348–0.9767; p < 0.001) (Fig 4B). Based on an optimal criterion value of 1.885, the diagnostic sensitivity was 76.92% with 100% of specificity (Youden’s index: 76.92%) for veal calves from trial 1 (S4 Table). Instead, sensitivity and specificity of the test on young bulls were respectively 53.57% and 100% using 1.820 as optimal criterion value (Youden’s index: 53.57%) (S5 Table).

The optimal criterion value selected was 1.885 with 76.92% sensitivity and 100% specificity and 1.820 with 53.57% sensitivity and 100% specificity for veal calves and young bulls respectively.

## Discussion

The presence of growth promoters in animals intended for human consumption is verified by official controls through chemical analytical investigations carried out on different animal matrices. Direct chemical analyses are sensitive and specific, but they present some drawbacks, including the lack of standards for new molecules’ identification, the rapid elimination of the drug and the high cost. Therefore, the development of new approaches, including direct and indirect methods of analysis, is promoted by the scientific community to support official methods of investigation [30].

Screening methods based on the recognition of the biological effects of growth promoters’ administration on animals produced encouraging results. These approaches allow the disclosure of illicit treatments even when the drug residues are not detectable by official analytical methods.

Blood parameter measurements such as serum oxidative stress factors [31,32] or selected plasma components [33] have been investigated and several candidate biomarkers have been proposed. Recent studies [34,35] proposed about 16 plasma proteins as potential biomarkers of treatment with low doses of dexamethasone: in particular, the sex hormone-binding globulin, histidine-rich glycoprotein and paraoxanase-1 showed significant differences in concentration after administration of dexamethasone in bovine.

Previous studies [36,37] exploited the ability of dexamethasone to affect cortisol metabolism and they proposed the 6β-hydroxycortisol/cortisol ratio in the urine as a useful indirect tool to identify dexamethasone treatment. Other approaches such as skeletal muscle gene expression profiling in bulls treated with dexamethasone were proposed [38,39]. In a previous study [23], an indirect screening method based on qPCR and detecting the up-regulation of oxytocin precursor gene in skeletal muscle of young bulls treated with dexamethasone and 17β-estradiol was developed.

The expression analysis of specific genes in tissue samples like thoracic thymus in beef cattle is a useful screening tool to evaluate the safety and quality of food products because it provides reliable results quickly and cost-effectively [40].

Usually, qPCR is the method of choice for evaluating the gene expression regulation, in particular using relative quantification method. However, the relative quantification requires a calibrator. On the other hand, absolute quantification is based on calibration curves that are built using known amounts of external calibrators [16]. ddPCR can be used for both absolute and relative quantification experiments but it does not require any standard curve [41].

Since the output of the analysis is given in copies of cDNA per microliters of reaction (with 95% confidence intervals), ddPCR system allows measurement of target gene expression with high precision calculated with intra- and inter-assay. In fact, the relative CV% values obtained using ddPCR technology were significantly lower than those calculated using qPCR in similar screening methods previously developed [23,42] in which mean intra- and inter- assay CV% were respectively 26.05% and 22.32% [23] and 20.91% and 19.66% [42].

*FKBP5* gene expression profile detected in this study by ddPCR in thoracic thymus was comparable to previous results obtained using qPCR technology [14], as well as specificity and sensitivity parameters described in analogue qPCR screening tests [23,42].

Anyway, the ROC curve for *FKBP5* down-regulation in GC treated veal calves showed a quite clear distinction between GC-treated and control animals with 76.92% of sensitivity and 100% of specificity confirming the capacity of the test to detect also prednisolone administration. In young bulls, the performance of the test was reduced and this is probably due to a weaker molecular response to the treatment in older animals.

The working range calculation was an important parameter to establish: in particular, we reported that exceeding the limit of 292 and 170 copies/μl for reaction for *FKBP5* and *TBP* respectively, is limiting for reagents concentration, in particular *FKBP5* primers. Therefore, we suggest to dilute the more concentrated samples. On the other hand, a concentration of cDNA up to 0.05 ng/μl, corresponding to 1 and 0.5 copies/μl of *FKBP5* and *TBP* respectively, allows to accurately determine the expression level of the target gene, since it is not affected by cDNA concentration.

The correlation coefficient values reflect the high linearity of the assay throughout different dilutions, allowing the detection of a wide range of transcript concentrations and low target copies. In addition, thanks to ddPCR technology, the detection limit of about 0.5–1 copies/ μl of cDNA was significantly lower than the detection limit obtained in a similar qPCR screening test (about 7–25 copies/ μl of cDNA) [23].

This ddPCR assay was developed as duplex amplification using EvaGreen dsDNA binding dye. The change in droplet amplitude relative to the two genes detected was obtained using different primers concentrations and different amplicons length. The correlation analysis between duplex and simplex mode is statistically significant allowing to apply the method as duplex form, reducing time and costs of the assay. Moreover, the analysis is faster since ddPCR do not require technical replicates unlike the screening tests previously described [23,43].

In conclusion, the measure of a biomarker using ddPCR and EvaGreen technology allowed the development of a robust and conceptually simple assay that could support the official methods of investigation in animal doping.

## Supporting information

S1 Fig Analysis of RGs in bovine thymus. young bulls. Expression stability values (M) of candidate RGs in the thymus of veal calves of trial 1 (A) and young bulls if trial 2 and 3 (B). The RG stability was performed using the geNorm algorithm.
(PDF)

S2 Fig 1D Droplet Plots of temperature gradient PCR from 65°C to 55°C. *TBP* amplicons and relative no template control (NTC) in A1-H1 and A2-H2 respectively; *FKBP5* amplicons and relative NTC in A7-H7 and A8-H8 respectively.
(PDF)

S3 Fig Linearity of dilution of *FKBP5* and *TBP* ddPCR duplex assay. Results are represented as mean and standard deviation of three replicate measurements of *FKBP5* (●), *TBP* (▲) and *FKBP5*/*TBP* (■) copies/μl versus dilution series starting from a known amount of cDNA. Linear regression for *FKBP5* and *TBP* amplification was calculated from 0.05 ng/μl to 25 ng/μl of cDNA.
(PDF)

S4 Fig In-field study. *FKBP5* (A) and *TBP* (B) copies/μl detected on 116 samples of thoracic thymus using duplex ddPCR. Dotted lines indicate the maximum and minimum values obtained from working interval previous determined.
(PDF)

S5 Fig Normalized target gene expression levels. Normalized target gene expression levels observed in DEX, PDN and K groups of trial 1 (A), trial 2 (B), and trial 3 (C) using ddPCR method. Results were represented as mean ratio of copies/μl of *FKBP5* and *TBP*. *** p < 0.001, ** p < 0.01, * p < 0.05.
(PDF)

S1 Table Primer details. RGs and target gene primer descriptions for gene expression analysis.
(DOCX)

S2 Table qPCR parameters. R^2^ values, slope, primer efficiencies, and Cq mean values of the RGs and *FKBP5* in the thoracic thymus of animals of trial 1 and trial 2 and 3.
(DOCX)

S3 Table Calibration curve parameters. Best-fit values of a calibration curve starting from triplicate of dilution series of a known amount of cDNA.
(DOCX)

S4 Table Veal calves ROC curve parameters. Criterion values express as normalized expression level (*FKBP5/TBP*) and coordinates of ROC curve for *FKBP5* down-regulation as a screening test to detect GC administration in veal calves.
(DOCX)

S5 Table Young bulls ROC curve parameters. Criterion values express as normalized expression level (*FKBP5/TBP*) and coordinates of ROC curve for *FKBP5* down-regulation as a screening test to detect GC administration in young bulls.
(DOCX)

S1 Dataset (ZIP)
