# Supplementary material for: Development of a droplet digital PCR assay to detect illicit glucocorticoid administration in bovine
Source: PLoS One. 2022 Jul 15;17(7):e0271613. doi: 10.1371/journal.pone.0271613 (PMC9286227; doi:10.1371/journal.pone.0271613)
Supplement: S1 Fig — Expression stability values (M) of candidate RGs in the thymus of veal calves of trial 1 (A) and young bulls if trial 2 and 3 (B). The RG stability was performed using the geNorm algorithm. (PDF) [file pone.0271613.s001.pdf]

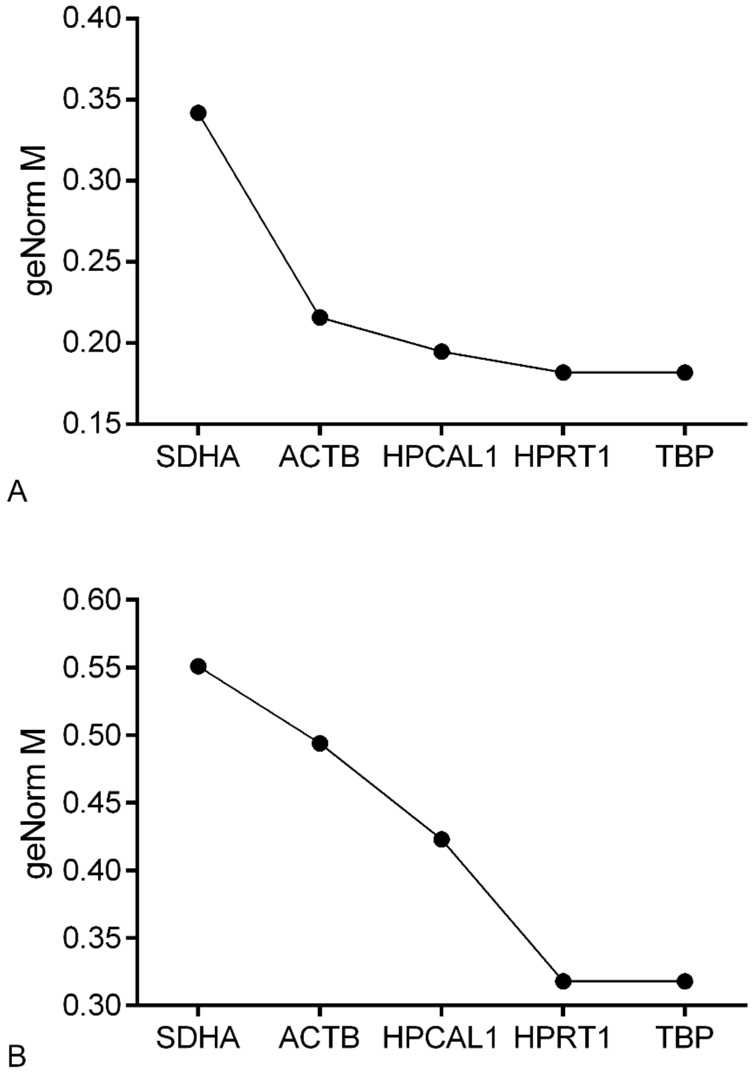

S1 figure. Analysis of RGs in bovine thymus. Expression stability values (M) of candidate RGs in the thymus of veal calves of trial 1 (A) and young bulls if trial 2 and 3 (B). The RG stability was performed using the geNorm algorithm
