# Supplementary material for: Development of a droplet digital PCR assay to detect illicit glucocorticoid administration in bovine
Source: PLoS One. 2022 Jul 15;17(7):e0271613. doi: 10.1371/journal.pone.0271613 (PMC9286227; doi:10.1371/journal.pone.0271613)
Supplement: S2 Fig — TBP amplicons and relative no template control (NTC) in A1-H1 and A2-H2 respectively; FKBP5 amplicons and relative NTC in A7-H7 and A8-H8 respectively. (PDF) [file pone.0271613.s002.pdf]

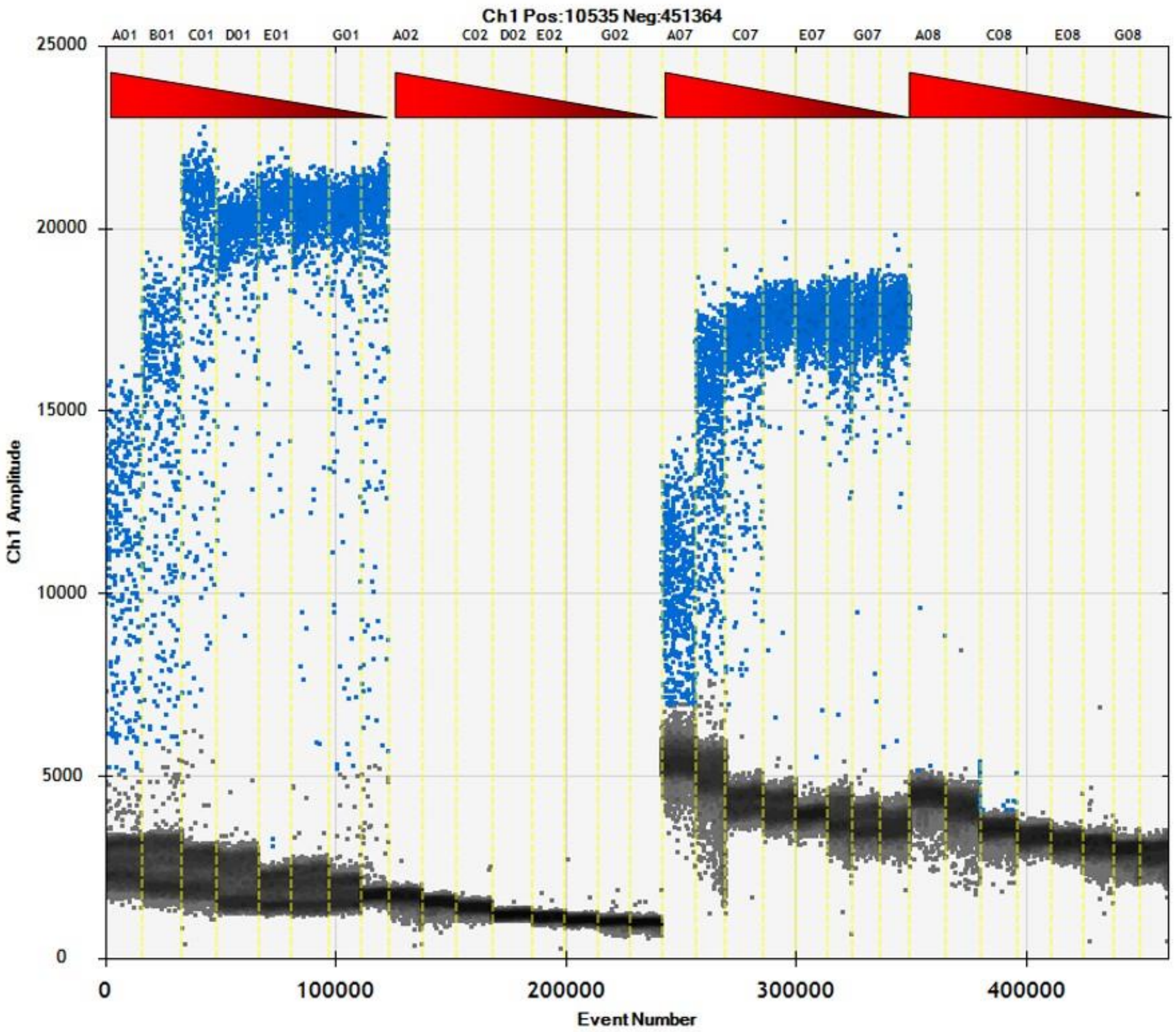

S2 figure. 1D Droplet Plots of temperature gradient PCR from 65°C to 55°C. TBP amplicons and relative no template control (NTC) in A1-H1 and A2-H2 respectively; FKBP5 amplicons and relative NTC in A7-H7 and A8-H8 respectively
