# Supplementary material for: Development of a droplet digital PCR assay to detect illicit glucocorticoid administration in bovine
Source: PLoS One. 2022 Jul 15;17(7):e0271613. doi: 10.1371/journal.pone.0271613 (PMC9286227; doi:10.1371/journal.pone.0271613)
Supplement: S3 Fig — Results are represented as mean and standard deviation of three replicate measurements of FKBP5 (●), TBP (▲) and FKBP5/TBP (■) copies/μl versus dilution series starting from a known amount of cDNA. Linear regression for FKBP5 and TBP amplification was calculated from 0.05 ng/μl to 25 ng/μl of cDNA. (PDF) [file pone.0271613.s003.pdf]

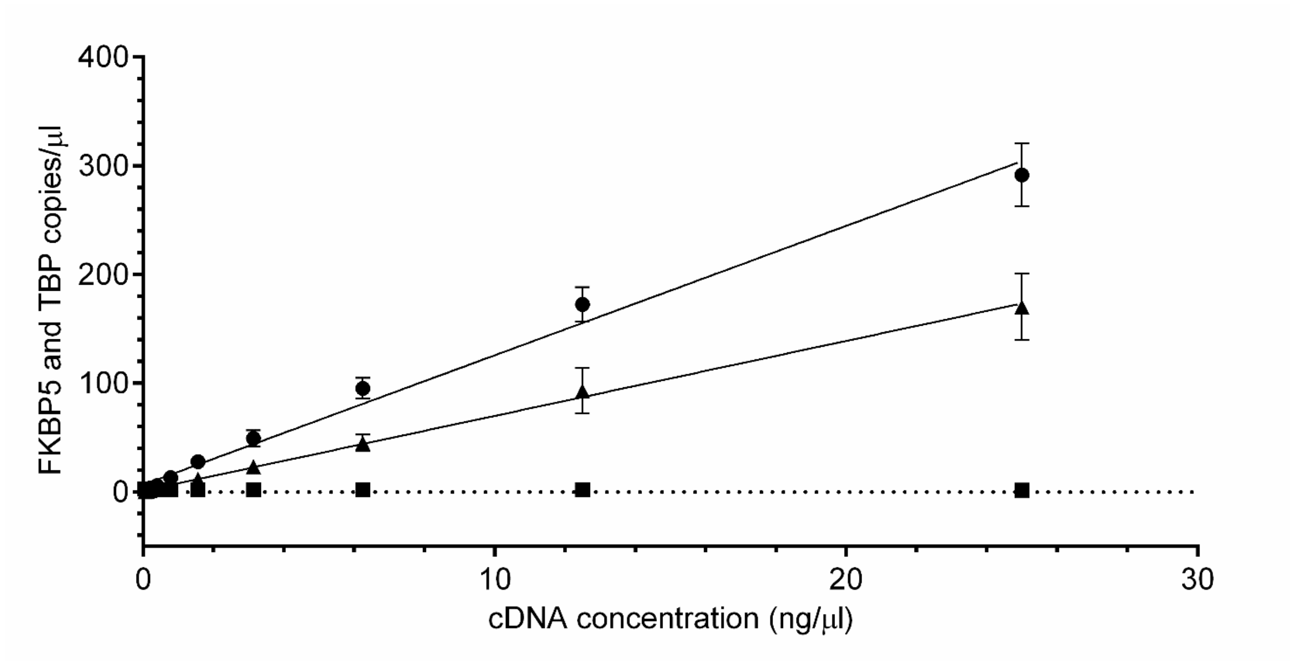

S3 figure. Linearity of dilution of FKBP5 and TBP ddPCR duplex assay. Results are represented as mean and standard deviation of three replicate measurements of *FKBP5* (●), TBP (▲) and FKBP5/TBP (■) copies/µl versus dilution series starting from a known amount of cDNA. Linear regression for FKBP5 and TBP amplification was calculated from 0.05 ng/µl to 25 ng/µl of cDNA.
