# Supplementary material for: Development of a droplet digital PCR assay to detect illicit glucocorticoid administration in bovine
Source: PLoS One. 2022 Jul 15;17(7):e0271613. doi: 10.1371/journal.pone.0271613 (PMC9286227; doi:10.1371/journal.pone.0271613)
Supplement: S4 Fig — FKBP5 (A) and TBP (B) copies/μl detected on 116 samples of thoracic thymus using duplex ddPCR. Dotted lines indicate the maximum and minimum values obtained from working interval previous determined. (PDF) [file pone.0271613.s004.pdf]

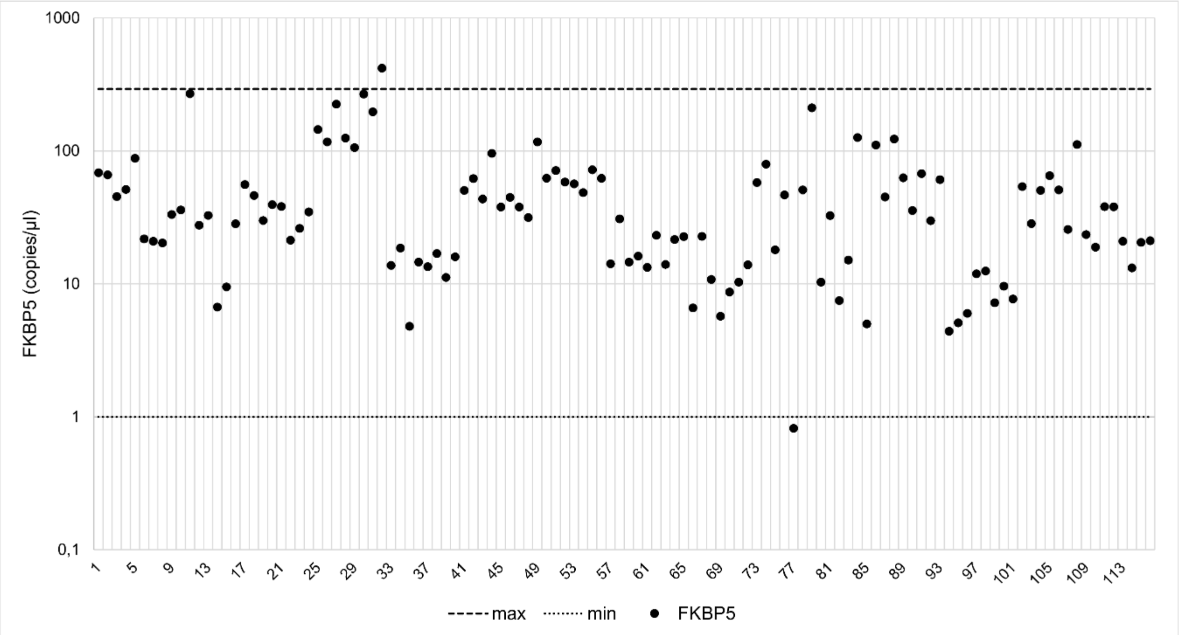

A

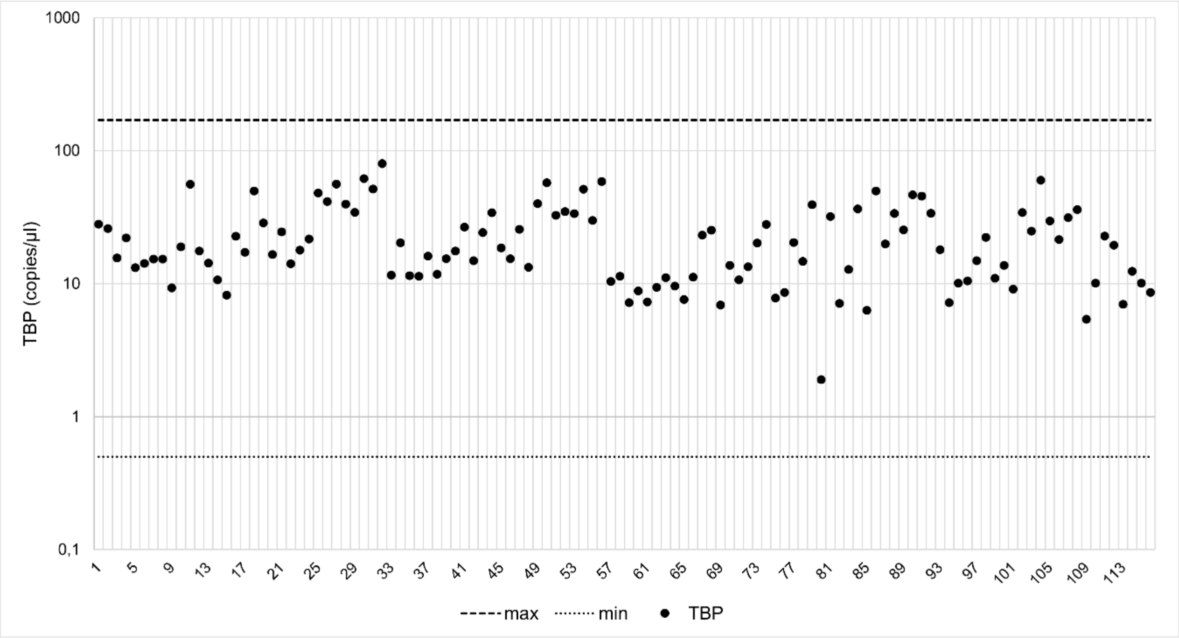

B

S4 figure. In-field study. *FKBP5* (A) and *TBP* (B) copies/µl detected on 116 samples of thoracic thymus using duplex ddPCR. Dotted lines indicate the maximum and minimum values obtained from working interval previous determined
