# Supplementary material for: Development of a droplet digital PCR assay to detect illicit glucocorticoid administration in bovine
Source: PLoS One. 2022 Jul 15;17(7):e0271613. doi: 10.1371/journal.pone.0271613 (PMC9286227; doi:10.1371/journal.pone.0271613)
Supplement: S5 Fig — Normalized target gene expression levels observed in DEX, PDN and K groups of trial 1 (A), trial 2 (B), and trial 3 (C) using ddPCR method. Results were represented as mean ratio of copies/μl of FKBP5 and TBP. *** p < 0.001, ** p < 0.01, * p < 0.05. (PDF) [file pone.0271613.s005.pdf]

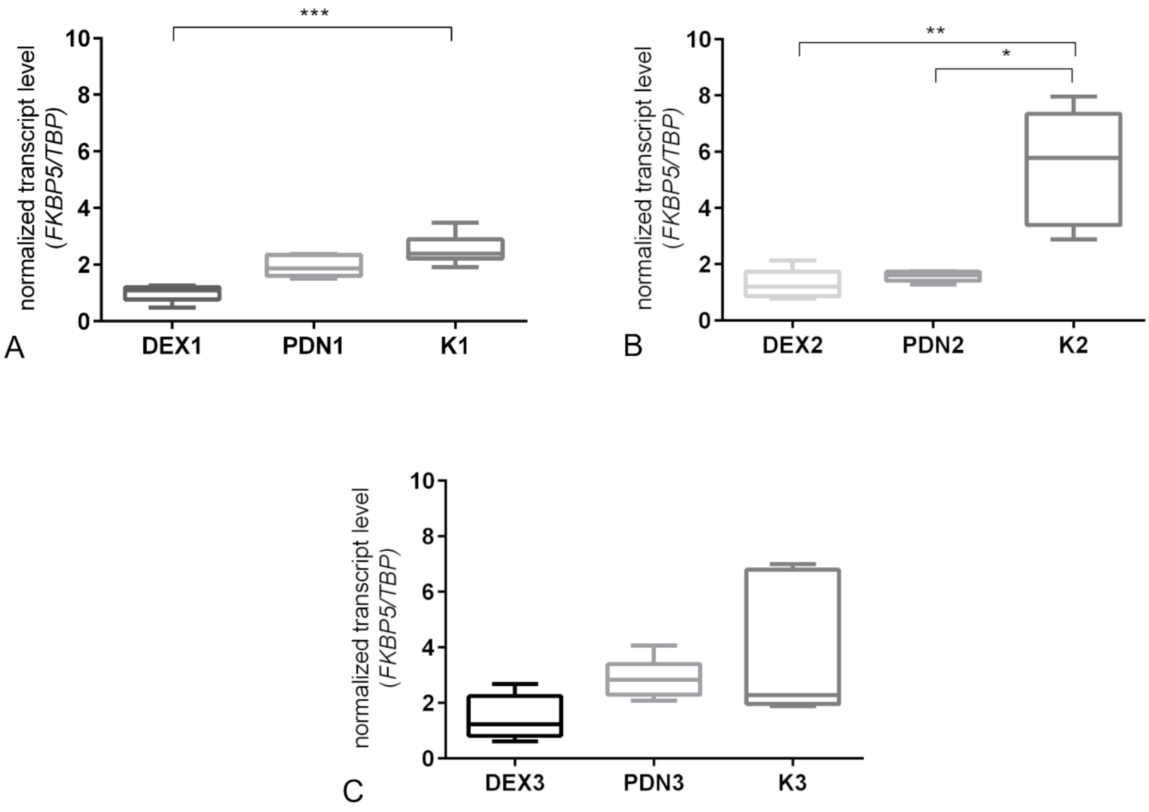

S5 figure. Normalized target gene expression level observed in DEX, PDN and K groups of trial 1 (A), trial 2 (B), and trial 3 (C) using ddPCR method. Results were represented as mean ratio of copies/µl of *FKBP5* and *TBP*. *** $p < 0.001$, ** $p < 0.01$, * $p < 0.05$.
