# Supplementary material for: Development of a droplet digital PCR assay to detect illicit glucocorticoid administration in bovine
Source: PLoS One. 2022 Jul 15;17(7):e0271613. doi: 10.1371/journal.pone.0271613 (PMC9286227; doi:10.1371/journal.pone.0271613)
Supplement: S1 Table — RGs and target gene primer descriptions for gene expression analysis. (DOCX) [file pone.0271613.s006.docx]

S1 Table. RGs and target gene primer descriptions for gene expression analysis

| Gene symbol (RefSeq) | Gene name | Biological process | Sequence (5’-3’) | Amplicon (bp) | Ref |
| --- | --- | --- | --- | --- | --- |
| *HPRT1* (NM_001034035.2) | hypoxanthine phosphoribosyl - transferase I | central role in the generation of purine nucleotides | F: TGCTGAGGATTTGGAGAAGG  R: CAACAGGTCGGCAAAGAACT | 154 | [1] |
| *ACTB* (NM_173979.3) | β-actin | structural constituent of cytoskeleton | F: CCCAGATCATGTTCGAGACC  R: GAGGCATACAGGGACAGCAC | 95 | [1] |
| *TBP*  (NM_001075742.1) | TATA-box binding protein | acrosome assembly | F: GAGAATAAGAGAGCCCCGCA  R: ACTTCACATCACAGCTCCCC | 191 | Primer-Blast |
| *HPCAL1*  (NM_001098964) | hippocalcin-like protein 1 | calcium ion binding | F: CCATCGACTTCAGGGAGTTC  R: CGTCGAGGTCATACATGCTG | 98 | [2] |
| *SDHA* (NM_174178.2) | succinate dehydrogenase complex, subunit A | tricarboxylic acid cycle | F: GACCAGGACGCCATCCATTA  R: CCCGAACTTGAGGCTCTGTC | 141 | Primer-Blast |
| *FKBP5*  (NM_001192862) | FKBP prolyl isomerase 5 | protein peptidyl-prolyl isomerization | F: CTGTGTACTTCAAGGGAGGCA  R: CAAGCAGAAAAGACTCGGAAGC | 126 | [3] |

1. Spalenza V, Girolami F, Bevilacqua C, Riondato F, Rasero R, Nebbia C, et al. Identification of internal control genes for quantitative expression analysis by real-time PCR in bovine peripheral lymphocytes. Vet J. 2011;189: 278–283. doi:10.1016/j.tvjl.2010.11.017

2. Saremi B, Sauerwein H, Dänicke S, Mielenz M. Technical note: Identification of reference genes for gene expression studies in different bovine tissues focusing on different fat depots. J Dairy Sci. 2012;95: 3131–3138. doi:10.3168/jds.2011-4803

3. Starvaggi Cucuzza L, Biolatti B, Scaglione FE, Cannizzo FT. Role of FKBP51 in the modulation of the expression of the corticosteroid receptors in bovine thymus following glucocorticoid administration. Domest Anim Endocrinol. 2018;62: 10–15. doi:10.1016/j.domaniend.2017.08.001
