# Supplementary material for: Development of a droplet digital PCR assay to detect illicit glucocorticoid administration in bovine
Source: PLoS One. 2022 Jul 15;17(7):e0271613. doi: 10.1371/journal.pone.0271613 (PMC9286227; doi:10.1371/journal.pone.0271613)
Supplement: S2 Table — R2 values, slope, primer efficiencies, and Cq mean values of the RGs and FKBP5 in the thoracic thymus of animals of trial 1 and trial 2 and 3. (DOCX) [file pone.0271613.s007.docx]

S2 Table. R^2^ values, slope, primer efficiencies, and Cq mean values of the RGs and FKBP5 in thoracic thymus of animals of trial 1 and trial 2 and 3

| Genes | Slope | R^2^ | Efficiency % | Amplificatory factor | Cq mean |
| --- | --- | --- | --- | --- | --- |
| Trial 1 (veal calves) | | | | | |
| *HPRT1* | -3.509 | 0.993 | 92.8 | 1.928 | 25.28 |
| *ACTB* | -3.141 | 0.998 | 108.2 | 2.082 | 22.52 |
| *TBP* | -3.17 | 0.992 | 106.7 | 2.067 | 26.22 |
| *SDHA* | -3.734 | 0.957 | 85.3 | 1.853 | 30.01 |
| *HPCAL1* | -3.012 | 0.948 | 114.8 | 2.148 | 29.73 |
| *FKBP5* | -3.239 | 0.997 | 103.6 | 2.036 | 26.02 |
| Trial 2 and 3 (young bulls) | | | | | |
| *HPRT1* | -3.203 | 0.996 | 105.2 | 2.052 | 26.15 |
| *ACTB* | -3.192 | 0.998 | 105.7 | 2.057 | 23.52 |
| *TBP* | -3.121 | 0.980 | 109.1 | 2.091 | 27.59 |
| *SDHA* | -3.592 | 0.981 | 89.8 | 1.898 | 30.69 |
| *HPCAL1* | -3.482 | 0.987 | 93.7 | 1.937 | 30.73 |
| *FKBP5* | -3.223 | 0.961 | 104.3 | 2.043 | 27.26 |
