# Supplementary material for: Development of a droplet digital PCR assay to detect illicit glucocorticoid administration in bovine
Source: PLoS One. 2022 Jul 15;17(7):e0271613. doi: 10.1371/journal.pone.0271613 (PMC9286227; doi:10.1371/journal.pone.0271613)
Supplement: S3 Table — Best-fit values of a calibration curve starting from triplicate of dilution series of a known amount of cDNA. (DOCX) [file pone.0271613.s008.docx]

S3 table. Best-fit values of a calibration curve starting from triplicate of dilution series of a known amount of cDNA.

|  | FKBP5 | TBP |
| --- | --- | --- |
| Best-fit values |  |  |
| Slope | 11,90 ± 0,4232 | 6,890 ± 0,1019 |
| Y-intercept when X=0.0 | 6,810 ± 3,863 | 1,215 ± 0,9300 |
| X-intercept when Y=0.0 | -0,5722 | -0,1764 |
| 1/slope | 0,08403 | 0,1451 |
| 95% Confidence Intervals |  |  |
| Slope | 10,92 to 12,88 | 6,655 to 7,125 |
| Y-intercept when X=0.0 | -2,099 to 15,72 | -0,9296 to 3,360 |
| X-intercept when Y=0.0 | -1,389 to 0,1689 | -0,4972 to 0,1325 |
| Goodness of Fit |  |  |
| R square | 0,9900 | 0,9983 |
| Sy.x | 10,22 | 2,462 |
| Is slope significantly non-zero? |  |  |
| F | 790,8 | 4574 |
| DFn, DFd | 1,000, 8,000 | 1,000, 8,000 |
| P value | < 0,0001 | < 0,0001 |
| Deviation from zero? | Significant | Significant |
| Data |  |  |
| Number of X values | 10 | 10 |
| Maximum number of Y replicates | 1 | 1 |
| Total number of values | 10 | 10 |
| Number of missing values | 0 | 0 |
|  |  |  |
| Equation | Y = 11,90*X + 6,810 | Y = 6,890*X + 1,215 |
