# Supplementary material for: Development of a droplet digital PCR assay to detect illicit glucocorticoid administration in bovine
Source: PLoS One. 2022 Jul 15;17(7):e0271613. doi: 10.1371/journal.pone.0271613 (PMC9286227; doi:10.1371/journal.pone.0271613)
Supplement: S4 Table — Criterion values express as normalized expression level (FKBP5/TBP) and coordinates of ROC curve for FKBP5 down-regulation as a screening test to detect GC administration in veal calves. (DOCX) [file pone.0271613.s009.docx]

S4 table. Criterion values express as normalized transcript level (FKBP5/TBP) and coordinates of ROC curve for FKBP5 down-regulation as screening test to detect GC administration in veal calves (confidence interval, CI; positive likelihood ratio, +LR; negative likelihood ratio, -LR; Youden’s index, J). The selected optimal criterion value is printed in bold.

| Criterion | Sensitivity% | 95% CI | Specificity% | 95% CI | +LR | -LR | J% |
| --- | --- | --- | --- | --- | --- | --- | --- |
| < 0.6750 | 7,692 | 0,1946 to 36,03 | 100 | 63,06 to 100,0 |  | 0,92 | 7,692 |
| < 0.9600 | 15,38 | 1,921 to 45,45 | 100 | 63,06 to 100,0 |  | 0,85 | 15,38 |
| < 1.090 | 23,08 | 5,038 to 53,81 | 100 | 63,06 to 100,0 |  | 0,77 | 23,08 |
| < 1.160 | 30,77 | 9,092 to 61,43 | 100 | 63,06 to 100,0 |  | 0,69 | 30,77 |
| < 1.220 | 38,46 | 13,86 to 68,42 | 100 | 63,06 to 100,0 |  | 0,62 | 38,46 |
| < 1.375 | 46,15 | 19,22 to 74,87 | 100 | 63,06 to 100,0 |  | 0,54 | 46,15 |
| < 1.550 | 53,85 | 25,13 to 80,78 | 100 | 63,06 to 100,0 |  | 0,46 | 53,85 |
| < 1.605 | 61,54 | 31,58 to 86,14 | 100 | 63,06 to 100,0 |  | 0,38 | 61,54 |
| < 1.735 | 69,23 | 38,57 to 90,91 | 100 | 63,06 to 100,0 |  | 0,31 | 69,23 |
| **< 1.885** | **76,92** | **46,19 to 94,96** | **100** | **63,06 to 100,0** |  | **0,23** | **76,92** |
| < 1.995 | 76,92 | 46,19 to 94,96 | 87,5 | 47,35 to 99,68 | 6,15 | 0,26 | 64,42 |
| < 2.125 | 84,62 | 54,55 to 98,08 | 87,5 | 47,35 to 99,68 | 6,77 | 0,18 | 72,12 |
| < 2.250 | 84,62 | 54,55 to 98,08 | 75 | 34,91 to 96,81 | 3,38 | 0,21 | 59,62 |
| < 2.340 | 84,62 | 54,55 to 98,08 | 62,5 | 24,49 to 91,48 | 2,26 | 0,25 | 47,12 |
| < 2.360 | 92,31 | 63,97 to 99,81 | 62,5 | 24,49 to 91,48 | 2,46 | 0,12 | 54,81 |
| < 2.375 | 100 | 75,29 to 100,0 | 62,5 | 24,49 to 91,48 | 2,67 |  | 62,5 |
| < 2.390 | 100 | 75,29 to 100,0 | 50 | 15,70 to 84,30 | 2,00 |  | 50 |
| < 2.625 | 100 | 75,29 to 100,0 | 37,5 | 8,523 to 75,51 | 1,60 |  | 37,5 |
| < 2.875 | 100 | 75,29 to 100,0 | 25 | 3,185 to 65,09 | 1,33 |  | 25 |
| < 3.190 | 100 | 75,29 to 100,0 | 12,5 | 0,3160 to 52,65 | 1,14 |  | 12,5 |
