# Supplementary material for: Development of a droplet digital PCR assay to detect illicit glucocorticoid administration in bovine
Source: PLoS One. 2022 Jul 15;17(7):e0271613. doi: 10.1371/journal.pone.0271613 (PMC9286227; doi:10.1371/journal.pone.0271613)
Supplement: S5 Table — Criterion values express as normalized expression level (FKBP5/TBP) and coordinates of ROC curve for FKBP5 down-regulation as a screening test to detect GC administration in young bulls. (DOCX) [file pone.0271613.s010.docx]

S5 table. Criterion values express as normalized transcript level (*FKBP5/TBP*) and coordinates of ROC curve for FKBP5 down-regulation as screening test to detect GC administration in young bulls (confidence interval, CI; positive likelihood ratio, +LR; negative likelihood ratio, -LR; Youden’s index, J). The selected optimal criterion value is printed in bold.

| criterion | Sensitivity% | 95% CI | Specificity% | 95% CI | +LR | -LR | J% |
| --- | --- | --- | --- | --- | --- | --- | --- |
| < 0.690 | 3,57 | 0,09 to 18,35 | 100 | 75,29 to 100,0 |  | 0,96 | 3,57 |
| < 0.770 | 7,14 | 0,88 to 23,50 | 100 | 75,29 to 100,0 |  | 0,93 | 7,14 |
| < 0.860 | 10,71 | 2,27 to 28,23 | 100 | 75,29 to 100,0 |  | 0,89 | 10,71 |
| < 0.975 | 14,29 | 4,03 to 32,67 | 100 | 75,29 to 100,0 |  | 0,86 | 14,29 |
| < 1.045 | 17,86 | 6,06 to 36,89 | 100 | 75,29 to 100,0 |  | 0,82 | 17,86 |
| < 1.140 | 21,43 | 8,30 to 40,95 | 100 | 75,29 to 100,0 |  | 0,79 | 21,43 |
| < 1.240 | 25 | 10,69 to 44,87 | 100 | 75,29 to 100,0 |  | 0,75 | 25 |
| < 1.305 | 28,57 | 13,22 to 48,67 | 100 | 75,29 to 100,0 |  | 0,71 | 28,57 |
| < 1.360 | 32,14 | 15,88 to 52,35 | 100 | 75,29 to 100,0 |  | 0,68 | 32,14 |
| < 1.460 | 35,71 | 18,64 to 55,93 | 100 | 75,29 to 100,0 |  | 0,64 | 35,71 |
| < 1.535 | 39,29 | 21,50 to 59,42 | 100 | 75,29 to 100,0 |  | 0,61 | 39,29 |
| < 1.575 | 42,86 | 24,46 to 62,82 | 100 | 75,29 to 100,0 |  | 0,57 | 42,86 |
| < 1.665 | 46,43 | 27,51 to 66,13 | 100 | 75,29 to 100,0 |  | 0,54 | 46,43 |
| < 1.735 | 50 | 30,65 to 69,35 | 100 | 75,29 to 100,0 |  | 0,50 | 50 |
| **< 1.820** | **53,57** | **33,87 to 72,49** | **100** | **75,29 to 100,0** |  | **0,46** | **53,57** |
| < 1.925 | 53,57 | 33,87 to 72,49 | 92,31 | 63,97 to 99,81 | 6,97 | 0,50 | 45,88 |
| < 2.000 | 53,57 | 33,87 to 72,49 | 84,62 | 54,55 to 98,08 | 3,48 | 0,55 | 38,19 |
| < 2.065 | 57,14 | 37,18 to 75,54 | 84,62 | 54,55 to 98,08 | 3,72 | 0,51 | 41,76 |
| < 2.115 | 60,71 | 40,58 to 78,50 | 84,62 | 54,55 to 98,08 | 3,95 | 0,46 | 45,33 |
| < 2.190 | 64,29 | 44,07 to 81,36 | 84,62 | 54,55 to 98,08 | 4,18 | 0,42 | 48,91 |
| < 2.260 | 64,29 | 44,07 to 81,36 | 76,92 | 46,19 to 94,96 | 2,79 | 0,46 | 41,21 |
| < 2.295 | 67,86 | 47,65 to 84,12 | 69,23 | 38,57 to 90,91 | 2,21 | 0,46 | 37,09 |
| < 2.320 | 71,43 | 51,33 to 86,78 | 69,23 | 38,57 to 90,91 | 2,32 | 0,41 | 40,66 |
| < 2.450 | 75 | 55,13 to 89,31 | 69,23 | 38,57 to 90,91 | 2,44 | 0,36 | 44,23 |
| < 2.630 | 78,57 | 59,05 to 91,70 | 69,23 | 38,57 to 90,91 | 2,55 | 0,31 | 47,8 |
| < 2.785 | 82,14 | 63,11 to 93,94 | 69,23 | 38,57 to 90,91 | 2,67 | 0,26 | 51,37 |
| < 2.985 | 82,14 | 63,11 to 93,94 | 61,54 | 31,58 to 86,14 | 2,14 | 0,29 | 43,68 |
| < 3.205 | 85,71 | 67,33 to 95,97 | 61,54 | 31,58 to 86,14 | 2,23 | 0,23 | 47,25 |
| < 3.355 | 89,29 | 71,77 to 97,73 | 61,54 | 31,58 to 86,14 | 2,32 | 0,17 | 50,83 |
| < 3.405 | 92,86 | 76,50 to 99,12 | 61,54 | 31,58 to 86,14 | 2,41 | 0,12 | 54,4 |
| < 3.495 | 96,43 | 81,65 to 99,91 | 61,54 | 31,58 to 86,14 | 2,51 | 0,06 | 57,97 |
| < 3.695 | 96,43 | 81,65 to 99,91 | 53,85 | 25,13 to 80,78 | 2,09 | 0,07 | 50,28 |
| < 3.945 | 96,43 | 81,65 to 99,91 | 46,15 | 19,22 to 74,87 | 1,79 | 0,08 | 42,58 |
| < 4.335 | 100 | 87,66 to 100,0 | 46,15 | 19,22 to 74,87 | 1,86 |  | 46,15 |
| < 5.700 | 100 | 87,66 to 100,0 | 38,46 | 13,86 to 68,42 | 1,62 |  | 38,46 |
| < 6.885 | 100 | 87,66 to 100,0 | 30,77 | 9,092 to 61,43 | 1,44 |  | 30,77 |
| < 6.985 | 100 | 87,66 to 100,0 | 23,08 | 5,038 to 53,81 | 1,30 |  | 23,08 |
| < 7.070 | 100 | 87,66 to 100,0 | 15,38 | 1,921 to 45,45 | 1,18 |  | 15,38 |
| < 7.555 | 100 | 87,66 to 100,0 | 7,692 | 0,1946 to 36,03 | 1,08 |  | 7,692 |
